# Entropy Generation in MHD Conjugate Flow with Wall Shear Stress over an Infinite Plate: Exact Analysis

**DOI:** 10.3390/e21040359

**Published:** 2019-04-03

**Authors:** Arshad Khan, Faizan ul Karim, Ilyas Khan, Tawfeeq Abdullah Alkanhal, Farhad Ali, Dolat Khan, Kottakkaran Sooppy Nisar

**Affiliations:** 1 Institute of Business and Management Sciences, the University of Agriculture, Peshawar 25000, Pakistan; 2 Department of Mathematics, City University of Science and Information Technology, Peshawar 25000, Pakistan; 3 Faculty of Mathematics and Statistics, Ton Duc Thang University, Ho Chi Minh City 72915, Vietnam; 4 Department of Mechatronics and System Engineering, College of Engineering, Majmaah University, Majmaah 11952, Saudi Arabia; 5 Department of Mathematics, College of Arts and Sciences Wadi Aldawaser, Prince Sattam bin Abdulaziz University, Wadi Aldawaser 11991, Saudi Arabia

**Keywords:** entropy generation, Bejan number, heat transfer, wall shear stress, ramped wall, porous medium

## Abstract

The current work will describe the entropy generation in an unsteady magnetohydrodynamic (MHD) flow with a combined influence of mass and heat transfer through a porous medium. It will consider the flow in the *XY* plane and the plate with isothermal and ramped wall temperature. The wall shear stress is also considered. The influences of different pertinent parameters on velocity, the Bejan number and on the total entropy generation number are reported graphically. Entropy generation in the fluid is controlled and reduced on the boundary by using wall shear stress. It is observed in this paper that by taking suitable values of pertinent parameters, the energy losses in the system can be minimized. These parameters are the Schmitt number, mass diffusion parameter, Prandtl number, Grashof number, magnetic parameter and modified Grashof number. These results will play an important role in the heat flow of uncertainty and must, therefore, be controlled and managed effectively.

## 1. Introduction

In compound responses the heat transfer procedure is dependably joined by the mass transfer progression. Perhaps it was expected that the investigation of joined heat and mass transfer is supportive to obtaining the best comprehension of various nominal transfer procedures. In a porous medium the convective heat transfer over a plate has numerous uses, for example, for nuclear reactors, oil production, thermal insulation systems and in separation processes in chemical engineering. Ranganathan and Viskanta [1] researched the boundary layer mixed convective fluid inserted in a porous medium over a vertical plate. They guaranteed that the viscus impacts are important and cannot be dismissed. The effect of thermal radiation and chemical reaction on the fluid with external heat source over a stretching surface was talked about by Mohan Krishna et al. [2], who observed that the chemical reaction parameter became important when the mass transfer rate was growing. Recently Gupta et al. [3] investigated the heat transfer for incompressible nanofluid over an inclined stretching sheet with a chemical reaction and radiation with the effect of an MHD mixed convective. Furthermore, Singh et al. [4] studied the computational approach for Jeffery–Hamel flow and Kumar et al. [5] studied the fractional model of convective radial fins with temperature-dependent thermal conductivity.

Scientists have been attempting to comprehend and diminish the challenges of industrial procedures to accomplish higher effectiveness. In engineering systems, there are different causes for entropy generation. In thermal systems, the primary source of entropy generation is mass transfer, heat transfer, viscous dissipation, coupling among heat, electrical conduction, mass transfer and chemical reaction, as examined in a pioneering series of publications by Bejan and co-workers [6,7]. At some exploratory examinations [8,9], the entropy growth standard has been used to decide the effectiveness of types of apparatus in numerous functional circumstances, for example, condensation, evaporation. The impact of magnetic field is seen again in a few human-made and natural streams. Magnetic fields are regularly connected in manufacturing to levitate heat, pump and mix fluid metals. There is the earthly magnetic field that is retained by fluid flow in the earth’s core, the sun powered magnetic field which creates sunspots and sun based flares, and the galactic magnetic field which is supposed to control the arrangement of stars from intergalactic mists. In recent times, significant consideration has been centered around uses of heat transfer and MHD, for example metallurgical handling, geothermal energy abstraction and MHD generators. The portent concerning MHD flow with mass and heat transfer is significant because of its various uses in innovation and science. The specific applications are set up in buoyancy-induced flows in the atmosphere, quasi-solid bodies and in forms of water, for example, earth. MHD flow with heat and mass transfer has obtained much attention in recent years because of their huge number of applications in engineering areas.

The handy utilization of the basics of heat and mass transfer in power system segments covers an extensive variety of essential designing systems, which incorporate pumps, heaters, turbines, compressors, cooling towers, heat transfer and so on. The utilization of the second law of thermodynamics to dissect fluid flow and heat in designing systems and devices has turned out to be progressively essential. The nonstop development of technology requires better cooling techniques and requests than enhanced heat transfer attributes. The studies on heat and mass transfer problems are also the hotspots in the heat transfer arena [10,11,12]. Bejan [13] studied the entropy generation minimizations [14] for the problems of heat and mass transfer and showed the classic uses in the intensive fields of thermal energy storage, heat transfer and mass transfer. Sahin reference [15] has explored entropy generation and the pumping power essential for a viscous laminar flow in a channel. Zhou et al. [16] additionally carried out optimization of a triangular SGR joint heat and mass transfer, and found that for the same volume in triangular model, the entropy generation is low than the rectangular generation. Recently Singh et al. [17] provided a numerical algorithm for the fractional Drinfeld–Sokolov–Wilson equation. Furthermore, the analytical techniques for system of time fractional nonlinear differential equations have been investigated by Choi et al. [18]. Furthermore, Awed [19] investigated a new definition of the Bejan number. The Bejan number is quite useful, as one can get evidence about the dominance of the magnetic field and fluid friction through heat transfer entropy or vice versa. Furthermore, the extending form of the Bejan number to a general form is investigated by Awad and Lage in reference [20]. Also the Hagen number versus Bejan number is investigated by Awad in reference [21]. Furthermore, the alternative form of the Darcy equation is studied in reference [22] and a review of entropy generation in micro channels is investigated in reference [23] by Awad.

The analysis of present literature shows that under a second law perspective, no work has yet been completed investigating flow over an infinite plate with wall shear stress and heat mass transfer. This problem needed to be studied from a second law point of view to calculate the performance of flows facing concentration difference, mass diffusion and loss in energy due to magnetic field and fluid friction. Therefore, the current effort is supported to get a better understanding of the damage to the handy energy faced in a conjugate flow together with wall shear stress, mass and heat transfer.

## 2. Flow Analysis

**Figure 1:**
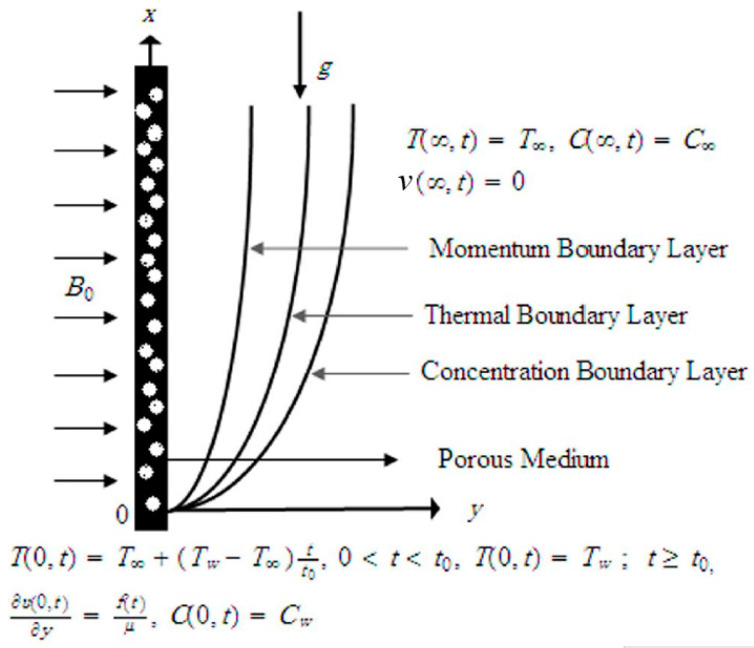
Physical configuration of the problem.

Assume the unsteady unidirectional MHD free convectional flow of an incompressible viscous fluid over a vertical unbounded plate. As shown in Figure 1, the x has been taken along the plate and y axis is perpendicular to the plate correspondingly. At the start, the fluid and plate both are at relaxation with $T_{W}$ (constant wall temperature). After a while, along the x axis the plate interrupts the fluid by time dependent shear stress f(t). Equivalently the temperature of the fluid is decreased or increased to $T_{\infty }\left(T_{W}+T_{\infty }\right)\frac{t}{t_{0}}$ when $t\leq t_{0}$ and afterwards for $t>t_{0}$ is continued at $T_{W}$. An unchanging magnetic field of strength $B_{0}$ is applied normal ally to the flow path. Considering the fluid is in the y>0 porous half space, the flow is laminar and ignoring the viscous dissipation by using Boussinesq’s approximation, by Butt et al. [24] the governing equations of the flow are given. The continuity, momentum, energy and concentration equations for the boundary layer flow are written as,

(1) $$\frac{\partial \Phi }{\partial x}+\frac{\partial \Phi }{\partial y}+\frac{\partial \Phi }{\partial z}=0,$$

(2) $$\frac{\partial \Phi }{\partial y}=\frac{1}{\rho }\frac{\partial \tau }{\partial y}+g\beta_{T}(T-T_{\infty })-\frac{\nu }{K}\Phi -\frac{\delta B_{{}^{\circ}}{}^{2}}{\rho }\Phi +g\beta_{C}(C-C_{\infty }),$$

(3) $$\frac{\partial \tau }{\partial y}=\mu \frac{\partial \Phi }{\partial y},$$

(4) $$\frac{\partial C}{\partial t}=D\frac{\partial^{2}C}{\partial y^{2}}.$$

First Law Analysis

(5) $$\rho c_{p}\frac{\partial T}{\partial t}=\kappa \frac{\partial^{2}T}{\partial y^{2}}-\frac{\partial q_{r}}{\partial y}.$$

Second Law Analysis

(6) $$S_{G}=\frac{\kappa }{T_{\infty }^{2}}{\left(\frac{\partial T}{\partial y}\right)}^{2}+\frac{\mu }{T_{\infty }}{\left(\frac{\partial \Phi }{\partial y}\right)}^{2}+\frac{\mu }{KT_{\infty }}\Phi^{2}+\frac{\sigma B_{0}^{2}}{T_{\infty }}\Phi^{2}+\frac{RD}{T_{\infty }}(\frac{\partial T}{\partial y})(\frac{\partial C}{\partial y})+\frac{RD}{C_{\infty }}{\left(\frac{\partial C}{\partial y}\right)}^{2}.$$

here Φ(y,t) in x direction is the fluid velocity, *B*_0_ the applied magnetic field, μ is the fluid viscosity, $q_{r}$ is Radiative heat flux, K permeability of porous medium, T(y,t) is the fluid temperature, v kinematic viscosity, $\beta_{T}$ coefficient of thermal expansion, g gravitational acceleration, non trivial shear stress is τ(y,t), σ electric conductivity of the fluid, $c_{p}$ heat capacity at constant pressure, k thermal conductivity, ρ constant density and $S_{G}$ volumetric rate of local entropy generation, *C* concentration, R the gas constant and *D* mass diffusivity are defined by Bejan [25].

The corresponding boundary and initial condition are as follows by assuming there are no slip acts in the middle of plate and fluid so,

(7) $$\begin{array}{l} \Phi (y,t)=0,\;\;T(y,0)=T_{\infty },\;\;C(y,0)=C_{\infty };\;\;\;\;\forall y\geq 0\\ T(0,\;t)\;=\;T_{W}\;\;t_{0}\geq 0\;\;\;\;∵t_{0}=\frac{\nu }{\Phi_{0}{}^{2}}\\ T(0,\;t)\;=\;T_{\infty }\;+\;(T_{W}-T_{\infty });\;\;\;\;\;0\;<\;t\;<\;t_{0}\\ \frac{\partial \Phi (0,t)}{\partial y}=\frac{f(t)}{\mu },\;\;C(0,t)=C_{W};\;\;\;\;t>0,\\ \Phi (\infty ,t)=0,\;\;T(\infty ,t)=T_{\infty },\;\;C(\infty ,t)=C_{\infty };\;\;\;\;\forall t>0. \end{array}$$

Under the Rosseland approximation for optically thick fluid [26,27] the radiation heat flux is given by,

(8) $$q_{r}=\frac{\Phi_{0}\sigma^{*}}{3K_{R}}\frac{\partial T^{4}}{\partial y}.$$

where $\sigma^{*}$ and $K_{R}$ are the Stefan-Boltzmann constant and the mean spectral absorption coefficient respectively. Considering that the temperature variance in the flow stays necessarily minor, at that point Equation (8) can be implemented by expanding to a linearized $T^{4}$ into a Taylor series around $T_{\infty }$ and ignoring higher order terms, we then have,

(9) $$T^{4}=4T_{\infty }{}^{3}T-3T_{\infty }{}^{4}.$$

Using Equation (9) into Equation (8) and substituting the achieved aftermath in Equation (5) we have,

(10) $$\mathrm{Pr}\frac{\partial T}{\partial t}=\nu (1+N_{r})\frac{\partial^{2}T}{\partial y^{2}};y,t>0$$

where Pr, v and Nr are defined by

(11) $$\mathrm{Pr}=\frac{\mu c_{p}}{k},\;\;Nr=\frac{16\sigma T_{\infty }{}^{3}}{3kk_{R}},\;\;v=\frac{\mu }{\rho }.$$

By familiarizing the following dimensionless variables

(12) $$\begin{array}{l} y^{*}=\frac{\Phi_{0}}{\nu }y,\;\;\Phi^{*}=\frac{\nu }{\Phi_{0}},\;\;t^{*}=\frac{\Phi_{0}{}^{2}}{\nu }t,\;\;C^{*}=\frac{C-C_{\infty }}{C_{W}-C_{\infty }}\\ f^{*}(t^{*})=\frac{\nu }{\Phi_{0}{}^{2}\mu }f(t_{0}t^{*}),\;\;T^{*}=\frac{T-T_{\infty }}{T_{W}-T_{\infty }},\;\;\tau^{*}=\frac{\tau }{\rho V^{2}} \end{array}$$

Into Equation (2), Equation (4) and Equation (10) also releasing the star notations, we get

(13) $$\frac{\partial \Phi }{\partial t}=\frac{\partial^{2}\Phi }{\partial y^{2}}-M\Phi -K_{p}\Phi +GrT+GmC,$$

(14) $${\mathrm{Pr}}_{eff}\frac{\partial T}{\partial t}=\frac{\partial^{2}T}{\partial y^{2}},$$

(15) $$\frac{\partial C}{\partial t}=\frac{1}{Sc}\frac{\partial^{2}C}{\partial y^{2}}.$$

where ${\mathrm{Pr}}_{eff}=\frac{\mathrm{Pr}}{1+Nr}$, is the effective prandlt number and

$$\begin{array}{l} M=\frac{\sigma \nu B_{0}}{\rho \Phi_{0}{}^{2}},\;\;t_{0}=\frac{\nu }{\Phi_{0}{}^{2}},\;\;K_{P}=\frac{\nu^{2}}{K\Phi_{0}{}^{2}},\;\;Gm=\frac{\nu g\beta_{C}(C_{W}-C_{\infty })}{\Phi_{0}{}^{3}},\\ Gr=\frac{\nu g\beta_{T}(T_{W}-T_{\infty })}{\Phi_{0}{}^{3}},\;\;Sc=\frac{\nu }{D} \end{array}$$

Are the magnetic parameter, the characteristic time, for the porous medium the inverse permeability parameter, modified Grashof number, the Grashof number and Schmidt number, correspondingly.

Equivalent dimensionless conditions are,

(16) $$\begin{array}{l} \Phi (y,t)=0,\;\;T(y,0)=T_{\infty },\;\;C(y,0)=0;\;\;\;\;\forall y\geq 0\\ T(0;\;t)\;=\;1;\;\;t>1,\;\;\;\;T(0;\;t)\;=t\;;\;\;\;\;\;0\;<\;t\;\leq 1\\ \frac{\partial \Phi (0,t)}{\partial y}=\frac{\nu f(t)}{\Phi_{0}{}^{2}\mu },\;\;C(0,t)=1;\;\;\;\;t>0,\\ \Phi (\infty ,t)=0,\;\;T(\infty ,t)=0,\;\;C(\infty ,t)=0;\;\;\;\;\forall t>0. \end{array}$$

## 3. Entropy Generation

For viscous fluid flow in a magnetic field the volumetric rate of local entropy generation $S_{G}$.

(17) $$S_{G}={\underline{\frac{\kappa }{T_{\infty }^{2}}{\left(\frac{\partial T}{\partial y}\right)}^{2}}}_{1}+{\underline{\frac{\sigma B_{0}^{2}}{T_{\infty }}\Phi^{2}}}_{2}+{\underline{\frac{RD}{T_{\infty }}(\frac{\partial T}{\partial y})(\frac{dC}{dy})+\frac{RD}{C_{\infty }}{\left(\frac{dC}{dy}\right)}^{2}}}_{3}+{\underline{\frac{\mu }{T_{\infty }}{\left(\frac{\partial \Phi v}{\partial y}\right)}^{2}+\frac{\mu }{KT_{\infty }}\Phi^{2}}}_{4}$$

where entropy generation due to heat transfer is 1, 2 is the entropy generation due to a magnetic field, 3 is entropy generation owed by mass transfer and 4 is entropy generation owed by fluid friction. Now applying dimensionless variables in Equation (17), we have,

(18) $$\begin{array}{l} S_{G}={\left(T_{W}-T_{\infty }\right)}^{2}\frac{\Phi_{0}{}^{2}\kappa }{\nu^{2}T_{\infty }^{2}}{\left(\frac{\partial T}{\partial y}\right)}^{2}+\frac{\sigma \;\Phi_{0}{}^{2}B_{0}^{2}}{T_{\infty }}\Phi^{2}+\frac{RD}{T_{\infty }}(\frac{\partial T}{\partial y})(\frac{dC}{dy})\\ +\frac{RD}{C_{\infty }}{\left(\frac{\partial C}{\partial y}\right)}^{2}+\frac{\Phi_{0}{}^{4}\mu }{T_{\infty }\nu^{2}}{\left(\frac{\partial \Phi }{\partial y}\right)}^{2}+\frac{\mu \Phi_{0}{}^{2}}{KT_{\infty }}\Phi^{2} \end{array}$$

Similarly

(19) $$N_{S}=\frac{S_{G}}{S_{0}}.$$

where $S_{0}$ is characteristic entropy generation rate, its value is

(20) $$S_{0}=\frac{\Phi_{0}{}^{2}\kappa }{T_{\infty }{}^{2}\nu^{2}}{\left(T_{W}-T_{\infty }\right)}^{2}$$

Using the value of $S_{0}$ in $S_{G}$, we get

(21) $$N_{S}={\left(\frac{\partial T}{\partial y}\right)}^{2}+\frac{B_{r}}{\Omega }{\left(\frac{\partial \Phi }{\partial y}\right)}^{2}+\frac{B_{r}K_{p}}{\Omega }\Phi^{2}+\frac{B_{r}M}{\Omega }\Phi^{2}+\frac{\lambda M_{d}}{\Omega^{2}}{\left(\frac{dC}{dy}\right)}^{2}+M_{d}\left(\frac{\partial T}{\partial y}\right)\left(\frac{dC}{dx}\right)$$

where λ is the concentration difference, $M_{d}$ is the mass diffusion parameter, Ω is the dimensionless temperature difference and the brinkman number is denoted by $B_{r}$, which are defined under,

$$\begin{array}{l} B_{r}=\frac{\mu V^{2}}{\kappa \left(T_{W}-T_{\infty }\right)},\;\Omega =\frac{T_{W}-T_{\infty }}{T_{\infty }},\\ M_{d}=\frac{RDT_{\infty }\left(C_{W}-C_{\infty }\right)}{\kappa \left(T_{W}-T_{\infty }\right)},\;\lambda =\frac{C_{W}-C_{\infty }}{C_{\infty }}. \end{array}$$

## 4. Solution of the Problem

To solve Equation (13) to Equation (15) under the conditions (16), by applying Laplace transform method and develop the below mentioned differential equations,

(22) $$\Phi^{\prime }(y,s)=\frac{\partial^{2}\Phi^{\prime }(y,s)}{\partial y^{2}}+GrT^{\prime }(y,s)+GmC^{\prime }\left(y,s\right)-K_{p}\Phi^{\prime }\left(y,s\right)-M\Phi^{\prime }\left(y,s\right),$$

(23) $$T^{\prime }\left(y,s\right)=\frac{1}{{\mathrm{Pr}}_{eff}s}\frac{\partial^{2}T^{\prime }\left(y,s\right)}{\partial y^{2}},$$

(24) $$C^{\prime }\left(y,s\right)=\frac{1}{S_{c}s}\frac{\partial^{2}C^{\prime }\left(y,s\right)}{\partial y^{2}}.$$

With boundary conditions,

(25) $$T(0,s)\;=\frac{1-e^{-s}}{s^{2}},\frac{\partial \Phi^{\prime }(0,s)}{\partial y}=F\left(s\right),C(0,s)=\frac{1}{s},\Phi (\infty ,s)=0,T(\infty ,s)=0,C(\infty ,s)=0.$$

Using Equation (25) in Equation (23) we get,

(26) $$T^{\prime }\left(y,s\right)=\frac{1}{s^{2}}e^{-y\sqrt{s{\mathrm{Pr}}_{eff}}}-\frac{e^{-s}}{s^{2}}e^{{}^{-y\sqrt{s{\mathrm{Pr}}_{eff}}}}.$$

Its inverse Laplace transform is

(27) T(y,t)=f(y,t)−f(y,t−1)H(t−1),

Here

(28) $$f\left(y,t\right)=\left(\frac{{\mathrm{Pr}}_{eff}y^{2}}{2}+t\right)erfc\left(\frac{\sqrt{{\mathrm{Pr}}_{eff}y}}{2\sqrt{t}}\right)-\frac{\sqrt{{\mathrm{Pr}}_{eff}t}}{\sqrt{\pi }}ye^{\left(\frac{-{\mathrm{Pr}}_{eff}y^{2}}{4t}\right)}.$$

Here *erfc*(.) is used for the complementary error function and *erf*(.) for error function of Gauss [15].

(29) $$\frac{\partial T\left(y,t\right)}{\partial y}{|}_{y=0}=\frac{2\sqrt{{\mathrm{Pr}}_{eff}}}{\sqrt{\pi }}\left(\sqrt{t}-\sqrt{t-1}H\left(t-1\right)\right),$$

Is the equivalent heat transfer rate called Nusselt number.

Now using Equation (25) in Equation (24) we get the solution in the form,

(30) $$C\left(y,s\right)=\frac{1}{s}e^{-y\sqrt{Sc\;s}},$$

its Laplace transform is in the form

(31) $$C\left(y,t\right)=erfc\left(\frac{y\sqrt{Sc}}{2\sqrt{t}}\right),$$

And

(32) $$\frac{\partial C\left(y,t\right)}{\partial y}{|}_{y=0}=-\frac{\sqrt{Sc}}{\sqrt{\pi t}}.$$

Is the equivalent Sherwood number or mass transfer rate.

Using Equation (25) in Equation (22), we have,

(33) $$\begin{array}{l} \Phi^{\prime }\left(y,s\right)=\frac{a_{0}\sqrt{s}}{s^{2}\left(s-a_{1}\right)\sqrt{s+H_{0}}}\exp\left(-y\sqrt{s+H_{0}}\right)-\frac{a_{0}\sqrt{s}\exp\left(-s\right)}{s^{2}\left(s-a_{1}\right)\sqrt{s+H_{0}}}\exp\left(-y\sqrt{s+H_{0}}\right)\\ -\frac{F\left(s\right)}{\sqrt{s+H_{0}}}\exp\left(-y\sqrt{s+H_{0}}\right)-\frac{a_{2}}{s^{2}(s-a_{1})}\exp\left(-y\sqrt{s{\mathrm{Pr}}_{eff}}\right)\\ +\frac{a_{2}\exp(-s)}{s^{2}(s-a_{1})}\exp\left(-y\sqrt{s{\mathrm{Pr}}_{eff}}\right)+\frac{a_{3}\sqrt{s}}{s\left(s-a_{4}\right)\sqrt{s+H_{0}}}\exp\left(-y\sqrt{s+H_{0}}\right)\\ -\frac{a_{5}}{s\left(s-a_{4}\right)}\exp\left(-y\sqrt{sSc}\right) \end{array}$$

Its corresponding Laplace inverse is,

(34) $$\Phi (y,t)=\Phi_{c}\left(y,t\right)+\Phi_{m}\left(y,t\right)$$

where

(35) $$\begin{array}{l} \Phi_{c}(y,t)=a_{0}\int_{0}^{t}\left(\frac{\exp\left(a_{1}\left(t-q\right)\right)erf\left(\sqrt{a_{1}\left(t-q\right)}\right)}{{\left(a_{1}\right)}^{\frac{3}{2}}}-\frac{2\sqrt{t-q}}{\sqrt{\pi }a_{1}}\right)\frac{\exp\left(-H_{0}q-\frac{y^{2}}{4q}\right)}{\sqrt{\pi q}}dq\\ +\left[\frac{a_{0}}{a_{1}\pi }\int_{0}^{t-1}\frac{\left(2\sqrt{t-1-q}\right)\exp\left(-H_{0}q-\frac{y^{2}}{4q}\right)}{\sqrt{q}}dq\right]H\left(t-1\right)\\ -\left[\frac{a_{0}}{{\left(a_{1}\right)}^{\frac{3}{2}}\sqrt{\pi }}\int_{0}^{t-1}\frac{erf\left(\sqrt{a_{1}\left(t-1-q\right)}\right)\exp\left(a_{1}\left(t-1-q\right)-H_{0}q-\frac{y^{2}}{4q}\right)}{\sqrt{q}}dq\right]H\left(t-1\right)\\ +\frac{a_{2}\exp\left(a_{1}\left(t-1\right)+y\sqrt{{\mathrm{Pr}}_{eff}a_{1}}\right)}{2a_{1}{}^{2}}erfc\left(\frac{y\sqrt{{\mathrm{Pr}}_{eff}}}{2\sqrt{t-1}}+\sqrt{a_{1\left(t-1\right)}}\right)H\left(t-1\right)\\ +\frac{a_{2}\exp\left(a_{1}\left(t-1\right)-y\sqrt{{\mathrm{Pr}}_{eff}a_{1}}\right)}{2a_{1}{}^{2}}erfc\left(\frac{y\sqrt{{\mathrm{Pr}}_{eff}}}{2\sqrt{t-1}}-\sqrt{a_{1\left(t-1\right)}}\right)H\left(t-1\right)\\ -\frac{a_{5}\exp\left(a_{4}t+y\sqrt{a_{4}Sc}\right)}{2a_{4}}erfc\left(\frac{y\sqrt{sc}}{2\sqrt{t}}+\sqrt{a_{4}t}\right)\\ -\frac{a_{5}\exp\left(a_{4}t-y\sqrt{a_{4}Sc}\right)}{2a_{4}}erfc\left(\frac{y\sqrt{sc}}{2\sqrt{t}}-\sqrt{a_{4}t}\right) \end{array}$$

And

(36) $$\Phi_{m}\left(y,t\right)=-\frac{1}{\sqrt{\pi }}\int_{0}^{t}\frac{f\left(t-q\right)\exp\left(-H_{0}q-\frac{y^{2}}{4q}\right)}{\sqrt{q}}dq$$

are the corresponding convective and mechanical parts of velocity.

Since Equations (27) and (35) this is noted that T(y,t) is correct for all +ve values of ${\mathrm{Pr}}_{eff}$ but for ${\mathrm{Pr}}_{eff}=1$, the convective part of velocity is not valid. Therefore, by putting ${\mathrm{Pr}}_{eff}=1$ in Equation (14) to get the convective part of velocity using same process we have the following result,

(37) $$\begin{array}{l} \Phi \left(y,t\right)=-\frac{2a_{13}}{\pi }\int_{0}^{t}\frac{\sqrt{t-q}\exp\left(-H_{0}q-\frac{y^{2}}{4q}\right)}{\sqrt{q}}dq\\ +\left(\frac{2a_{13}}{\pi }\int_{0}^{t-1}\frac{\sqrt{t-1-q}\exp\left(-H_{0}q-\frac{y^{2}}{4q}\right)}{\sqrt{q}}dq\right)\\ +a_{3}\int_{0}^{t}\left(\frac{\exp\left(a_{4}\left(t-q\right)\right)erf\left(\sqrt{a_{4}\left(t-q\right)}\right)}{\sqrt{a_{4}}}-\frac{2\sqrt{t-q}}{\sqrt{\pi }a_{1}}\right)\frac{\exp\left(-H_{0}q-\frac{y^{2}}{4q}\right)}{\sqrt{\pi q}}dq\\ +a_{13}\left[\left(t+\frac{y^{2}}{2}\right)erfc\left(\frac{y}{2\sqrt{t}}\right)-\frac{y\sqrt{t}}{\sqrt{\pi }}\exp\left(\frac{-y^{2}}{4t}\right)\right]\\ -\frac{a_{5}\exp\left(a_{4}t-y\sqrt{a_{4}Sc}\right)}{\sqrt{a_{4}}}erfc\left(\frac{y\sqrt{Sc}}{2\sqrt{t}}-\sqrt{a_{4}t}\right)\\ -a_{13}\left[\left(\left(t-1\right)+\frac{y^{2}}{2}\right)erfc\left(\frac{y}{2\sqrt{t-1}}\right)-\frac{y\sqrt{t-1}}{\sqrt{\pi }}\exp\left(\frac{-y^{2}}{4\left(t-1\right)}\right)\right]H\left(t-1\right)\\ +\frac{a_{5}}{a_{4}}erfc\left(\frac{y\sqrt{Sc}}{2\sqrt{t}}\right)-\frac{1}{\sqrt{\pi }}\int_{0}^{t}\frac{f\left(t-q\right)\exp\left(-H_{0}q-\frac{y^{2}}{4q}\right)}{\sqrt{q}}dq\\ -\frac{a_{5}\exp\left(a_{4}t+y\sqrt{a_{4}Sc}\right)}{2a_{4}}erfc\left(\frac{y\sqrt{Sc}}{2\sqrt{t}}+\sqrt{a_{4}t}\right). \end{array}$$

### Constant Temperature on the Plate

Near the isothermal plate, the velocity and rate of heat transfer can be show for the flow as,

(38) $$T\left(y,t\right)=erfc\left(\frac{\sqrt{{\mathrm{Pr}}_{eff}y}}{2\sqrt{t}}\right)$$

(39) $$\frac{\partial T\left(y,t\right)}{\partial y}{|}_{y=0}=\frac{\sqrt{{\mathrm{Pr}}_{eff}}}{\sqrt{\pi t}}$$

(40) $$\begin{array}{l} \Phi_{c}(y,t)=\frac{a_{0}}{\sqrt{\pi a_{1}}}\int_{0}^{t}\frac{\exp\left(a_{1}\left(t-q\right)-H_{0}q-\frac{y^{2}}{4q}\right)erf\left(\sqrt{a_{1}\left(t-q\right)}\right)}{\sqrt{q}}dq\\ +\frac{a_{3}}{\sqrt{\pi a_{4}}}\int_{0}^{t}\frac{\exp\left(a_{4}\left(t-q\right)-H_{0}q-\frac{y^{2}}{4q}\right)erf\left(\sqrt{a_{4}\left(t-q\right)}\right)}{\sqrt{q}}dq\\ -\frac{a_{2}\exp\left(a_{1}t+y\sqrt{{\mathrm{Pr}}_{eff}a_{1}}\right)}{2a_{1}}erfc\left(\frac{y\sqrt{{\mathrm{Pr}}_{eff}}}{2\sqrt{t}}+\sqrt{a_{1}t}\right)\\ -\frac{a_{2}\exp\left(a_{1}t-y\sqrt{{\mathrm{Pr}}_{eff}a_{1}}\right)}{2a_{1}}erfc\left(\frac{y\sqrt{{\mathrm{Pr}}_{eff}}}{2\sqrt{t}}-\sqrt{a_{1}t}\right)\\ +\frac{a_{5}\exp\left(a_{4}t+y\sqrt{a_{4}Sc}\right)}{2a_{4}}erfc\left(\frac{y\sqrt{sc}}{2\sqrt{t}}+\sqrt{a_{4}t}\right)\\ --\frac{a_{5}\exp\left(a_{4}t-y\sqrt{a_{4}Sc}\right)}{2a_{4}}erfc\left(\frac{y\sqrt{sc}}{2\sqrt{t}}-\sqrt{a_{4}t}\right)\\ +\frac{a_{2}}{a_{1}}erfc\left(\frac{y\sqrt{{\mathrm{Pr}}_{eff}}}{2\sqrt{t}}\right)+\frac{a_{5}}{a_{4}}erfc\left(\frac{y\sqrt{Sc}}{2\sqrt{t}}\right), \end{array}$$

(41) $$\Phi_{m}\left(y,t\right)=-\frac{1}{\sqrt{\pi }}\int_{0}^{t}\frac{f\left(t-q\right)\exp\left(-H_{0}q-\frac{y^{2}}{4q}\right)}{\sqrt{q}}dq.$$

Equation (40) is not effective for ${\mathrm{Pr}}_{eff}=1$, thus by taking ${\mathrm{Pr}}_{eff}=1$ in Equation (14) and assuming a similar technique, we get:

(42) $$\begin{array}{l} \Phi \left(y,t\right)=a_{13}erfc\left(\frac{y}{2\sqrt{t}}\right)-\frac{a_{13}}{\sqrt{\pi }}\int_{0}^{t}\frac{\exp\left(-H_{0}q-\frac{y^{2}}{4q}\right)}{\sqrt{\left(t-q\right)q}}\\ +\frac{a_{3}}{\sqrt{\pi a_{4}}}\int_{0}^{t}\frac{\exp\left(a_{4}\left(t-q\right)-H_{0}q-\frac{y^{2}}{4q}\right)erf\left(\sqrt{a_{4}\left(t-q\right)}\right)}{\sqrt{q}}dq\\ +\frac{a_{5}}{a_{4}}erfc\left(\frac{y\sqrt{Sc}}{2\sqrt{t}}\right)-\frac{1}{\sqrt{\pi }}\int_{0}^{t}\frac{f\left(t-q\right)\exp\left(-H_{0}q-\frac{y^{2}}{4q}\right)}{\sqrt{q}}dq\\ -\frac{a_{5}}{2a_{4}}\exp\left(a_{4}t+y\sqrt{a_{4}Sc}\right)erfc\left(\frac{y\sqrt{Sc}}{2\sqrt{t}}+\sqrt{a_{4}t}\right)\\ -\frac{a_{5}}{2a_{4}}\exp\left(a_{4}t-y\sqrt{a_{4}Sc}\right)erfc\left(\frac{y\sqrt{Sc}}{2\sqrt{t}}-\sqrt{a_{4}t}\right) \end{array}$$

(43) $$\begin{array}{l} a_{0}=\frac{Gr\sqrt{{\mathrm{Pr}}_{eff}}}{{\mathrm{Pr}}_{eff}-1},a_{1}=\frac{H_{0}}{{\mathrm{Pr}}_{eff}-1},a_{2}=\frac{Gr}{{\mathrm{Pr}}_{eff}-1},\\ a_{3}=\frac{Gm\sqrt{Sc}}{Sc-1},a_{4}=\frac{H_{0}}{Sc-1},a_{5}=\frac{Gm}{Sc-1},a_{6}=\frac{Gr\sqrt{\mathrm{Pr}}}{\mathrm{Pr}-1},\\ a_{7}=\frac{H_{0}}{\mathrm{Pr}-1},a_{8}=\frac{Gr}{\mathrm{Pr}-1},a_{9}=\frac{K_{p}}{{\mathrm{Pr}}_{eff}-1},a_{10}=\frac{K_{p}}{{\mathrm{Pr}}_{eff}-1},\\ a_{11}=\frac{M}{{\mathrm{Pr}}_{eff}-1},a_{12}=\frac{M}{Sc-1},a_{13}=\frac{Gr}{H_{0}},H_{0}=K_{p}+M. \end{array}$$

## 5. Special Cases

There is a more general form of the velocity in Section 4. Therefore, to distinguish the physical understanding of the problem, we will show some special cases for its solutions with limiting solutions. Therefore, we discuss some special cases for ramped and isothermal plate, as in the literature its technical applicability is well known. As discussed in several books and articles, initially *shear stress is* produced due to friction between *fluid* particles and due to *fluid* viscosity. Indeed, *fluids* at rest cannot resist a *shear stress*; i.e., when a *shear stress* is applied to the static *fluid*, the *fluid* will not remain at rest, but will move because of the *shear stress. This idea of shear stress and in particular for the accelerating and arbitrary shear stresses as discussed in the following two cases, where the traditional shear* failure always occurs. Particularly, in high temperature in a high grade asphalt pavement, *accelerating or arbitrary shear stress is needed.*

### 5.1. Case-1

Here we consider $f\left(t\right)=ft^{b}\left(b>0\right)$ where the plate put on an accelerating shear stress to the fluid and so the mechanical part becomes,

(44) $$\Phi_{m}\left(y,t\right)=-\frac{f}{\sqrt{\pi }}\int_{0}^{t}\frac{{\left(t-q\right)}^{b}\exp\left(-H_{0}q-\frac{y^{2}}{4q}\right)}{\sqrt{q}}dq.$$

For M=0 the equivalent result is,

(45) $$\Phi_{m}\left(y,t\right)=-\frac{f}{\sqrt{\pi }}\int_{0}^{t}\frac{{\left(t-q\right)}^{b}\exp\left(-Kpq-\frac{y^{2}}{4q}\right)}{\sqrt{q}}dq.$$

Which is the same as with Corina et al. [19] (Equation (32).

Furthermore, when $K_{p}=0$, Equation (45) gives

(46) $$\Phi_{m}\left(y,t\right)=-\frac{f}{\sqrt{\pi }}\int_{0}^{t}\frac{{\left(t-q\right)}^{b}\exp\left(-\frac{y^{2}}{4q}\right)}{\sqrt{q}}dq.$$

### 5.2. Case-2

Here we taking the arbitrary function f(t)=fH(t), where *H*(.) is used for unit step function and f is a dimensionless constant. The shear stress is applied to the fluid after some time. The convective mechanical part of the velocity becomes as follows

(47) $$\Phi_{m}\left(y,t\right)=-\frac{f}{\sqrt{\pi }}\int_{0}^{t}\frac{\exp\left(-H_{0}q-\frac{y^{2}}{4q}\right)}{\sqrt{q}}dq,$$

For $K_{p}\neq 0,M\neq 0$ equivalently

(48) $$\Phi_{m}\left(y,t\right)=-\frac{f}{\sqrt{H_{0}}}\exp\left(-y\sqrt{H_{0}}\right)+\frac{2f}{\sqrt{\pi }}\int_{\sqrt{t}}^{\infty }\exp\left(-H_{0}z^{2}-\frac{y^{2}}{4z^{2}}\right)dz.$$

Moreover, if we put M=0 in Equation (47) we have

(49) $$\Phi_{m}\left(y,t\right)=-\frac{f}{\sqrt{Kp}}\exp\left(-y\sqrt{Kp}\right)+\frac{2f}{\sqrt{\pi }}\int_{\sqrt{t}}^{\infty }\exp\left(-Kpz^{2}-\frac{y^{2}}{4z^{2}}\right)dz.$$

Which is quite the same as Corina et al. [28] (Equation (28)) with the modification of $\sqrt{K_{p}}$

Now if we take both $K_{p}=0$ and M=0, Equation (47) has the form

(50) $$\Phi_{m}\left(y,t\right)=-\frac{f}{\sqrt{\pi }}\int_{0}^{t}\frac{\exp\left(-\frac{y^{2}}{4q}\right)}{\sqrt{q}}dq.$$

## 6. Results and Discussion

To analyze the physical understanding and the flow behavior of the results taken from dimensionless velocity, temperature and the corresponding irreversibility analysis, a chain of numerical calculation has taken out for several values of embedded parameters.

### 6.1. The Effects on Velocity

**Figure 2:**
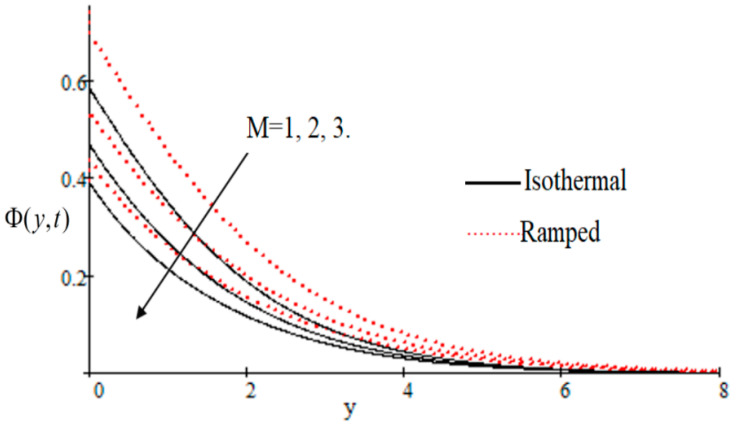
Velocity profile effecting by M.

**Figure 3:**
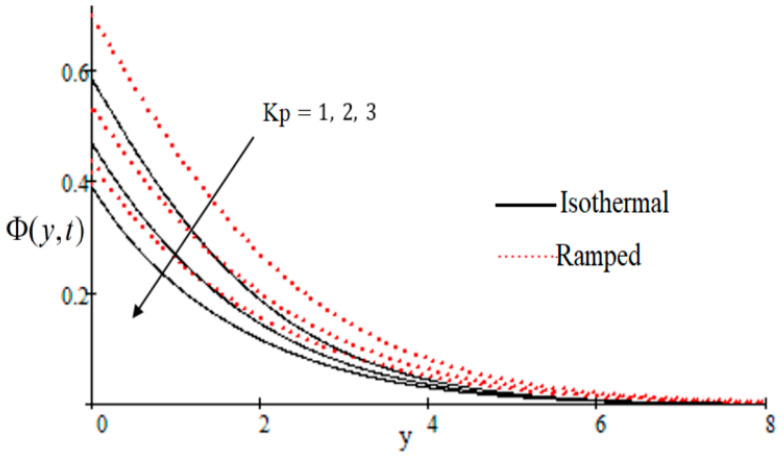
Influence of Kp on velocity profile.

**Figure 4:**
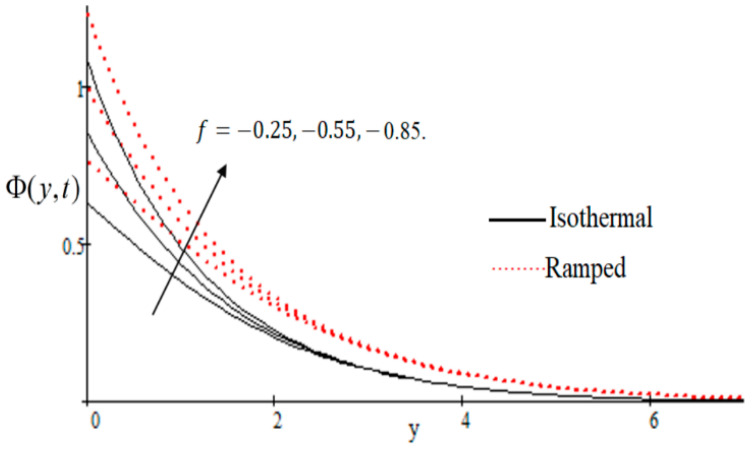
Velocity profile effecting by wall shear stress f.

**Figure 5:**
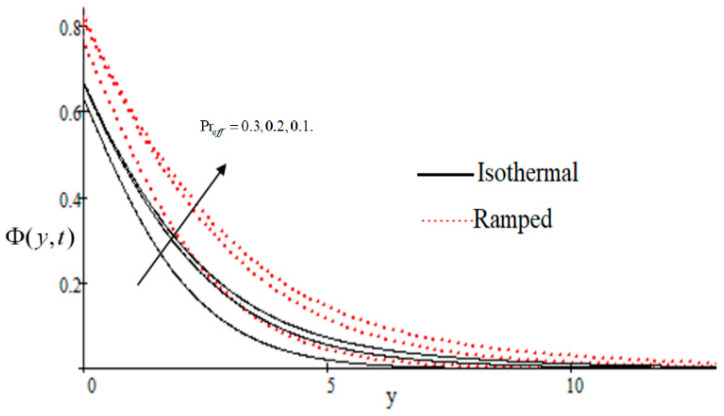
Influence of effective Prandlt number ${\mathrm{Pr}}_{eff}$ on velocity profile.

**Figure 6:**
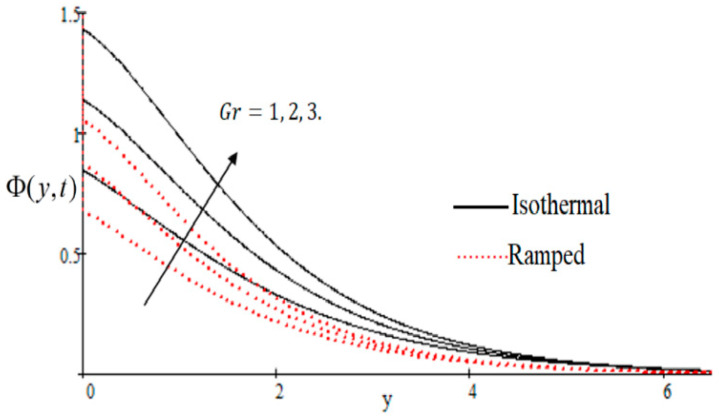
Influence of Gr on velocity profile.

**Figure 7:**
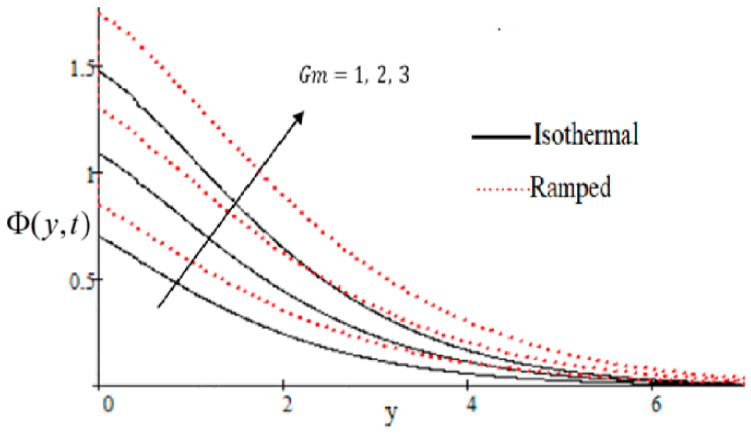
Gm effecting velocity profile.

**Figure 8:**
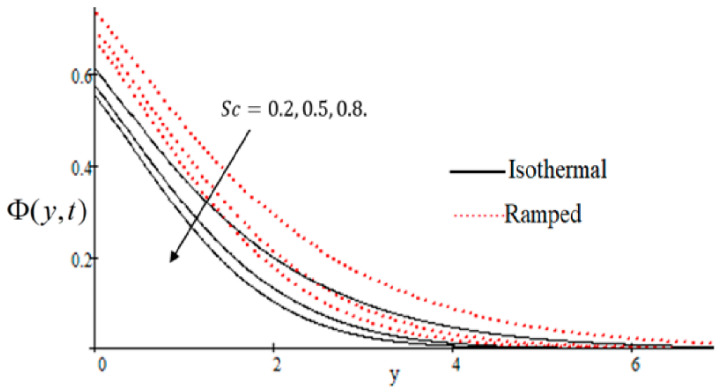
Velocity profile effecting by Sc.

In Figure 2, it has perceived that the velocity profile is reducing with increasing *M* in both ramped and isothermal wall temperature situations. Actually, it is because of an increase in magnetic field *M* which leads the frictional force to decrease and causes it to resist the flow of the fluid, therefore velocity decreases. The effect of Kp inverse permeability parameter over isothermal and ramped walls have been seen in Figure 3. It is noticed from the graph that velocity is decreasing with an increase in Kp. This effect occurs due to the porous medium, which is an increasing Kp that strengthens the resistance and consequently decreases the velocity. The influence of shear stress f is showed in Figure 4 where we noticed that when the value of f decreases, the velocity of fluid increases. In Figure 5 it is shown that when the value of effective prandlt number ${\mathrm{Pr}}_{eff}$ is decreased, the velocity increases for both isothermal and ramped walls. The influence of Grashof number Gr on the velocity is shown in Figure 6, where it has observed that velocity increase with increasing Gr. The influence of modified Grashof number Gm on the velocity is also increasing, it increases the velocity more rapidly than Gr showed in Figure 7. The effect of Schmidt number Sc is shown in Figure 8. It is detected that by increasing the value of Sc, the velocity decreases.

### 6.2. Mechanism of $N_{S}$ by Different Parameters

**Figure 9:**
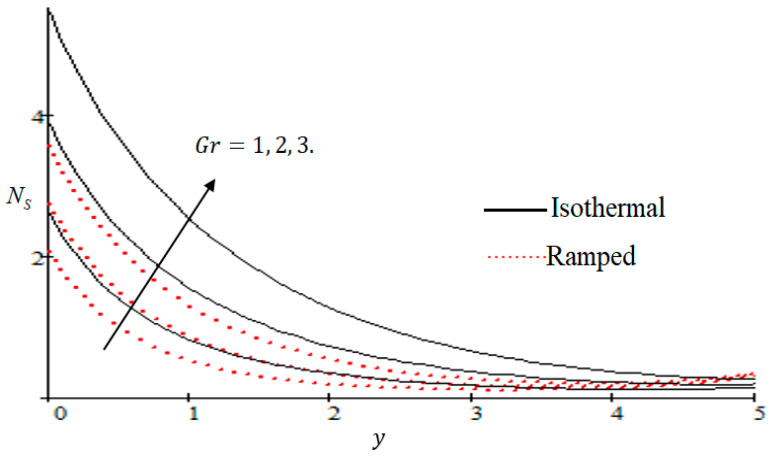
Influence of Gr on Ns.

**Figure 10:**
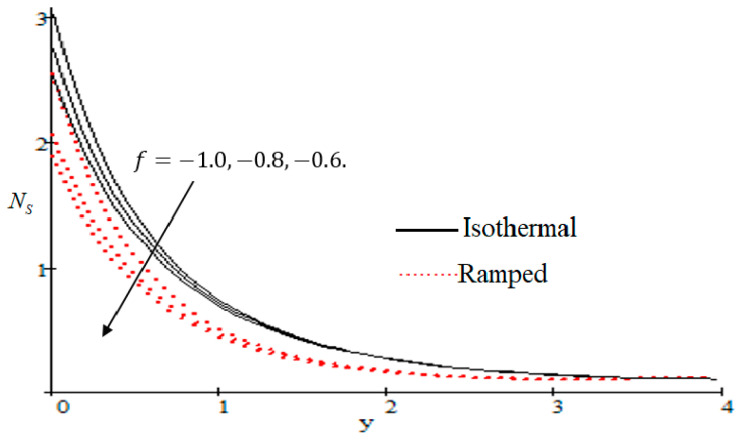
Influence of f on Ns.

**Figure 11:**
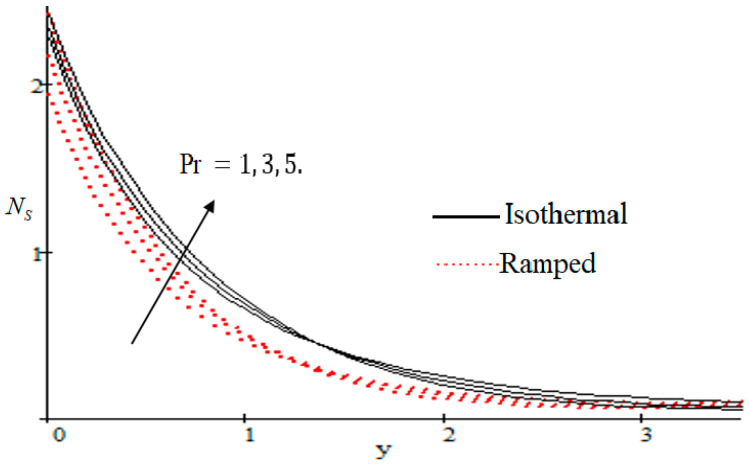
Influence of ${\mathrm{Pr}}_{eff}$ on Ns.

**Figure 12:**
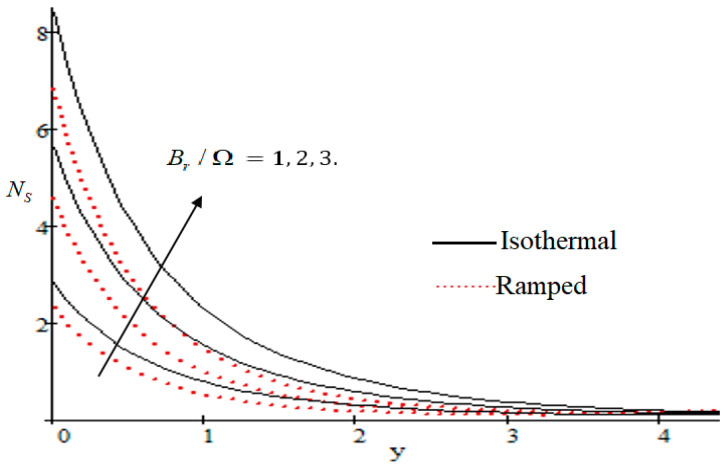
Influence of $B_{r}/\Omega$ on Ns.

**Figure 13:**
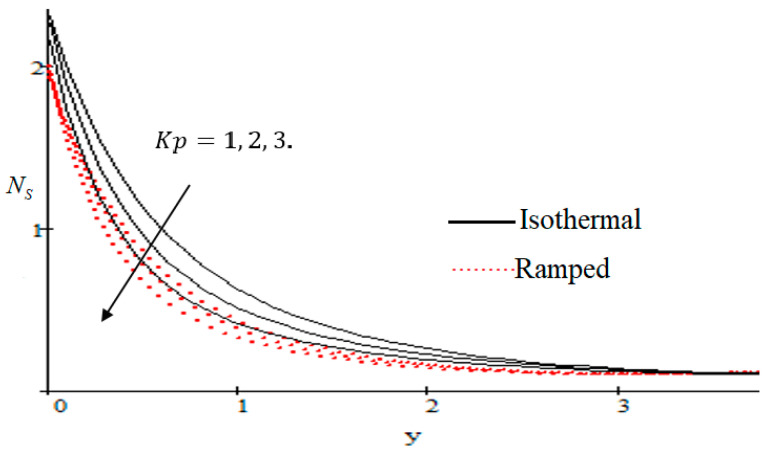
Influence of $K_{p}$ on Ns.

**Figure 14:**
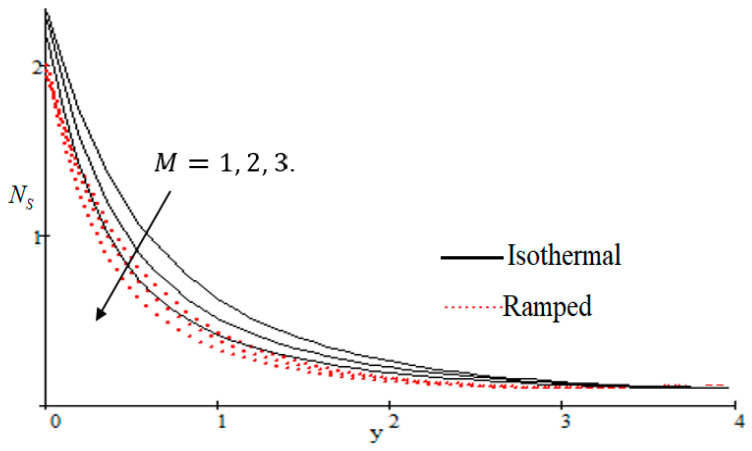
Influence of M on Ns.

**Figure 15:**
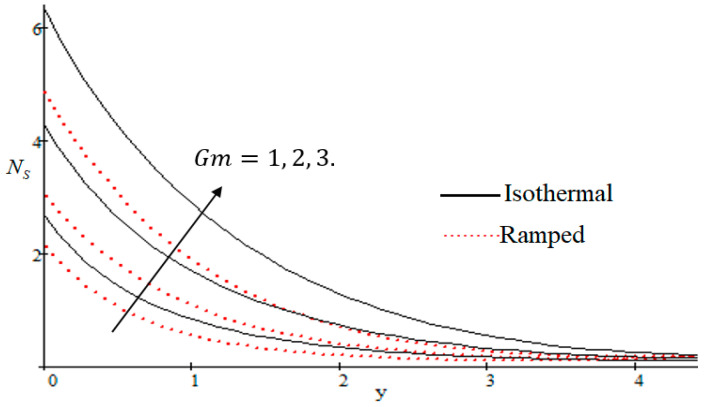
Influence of Gm on Ns.

**Figure 16:**
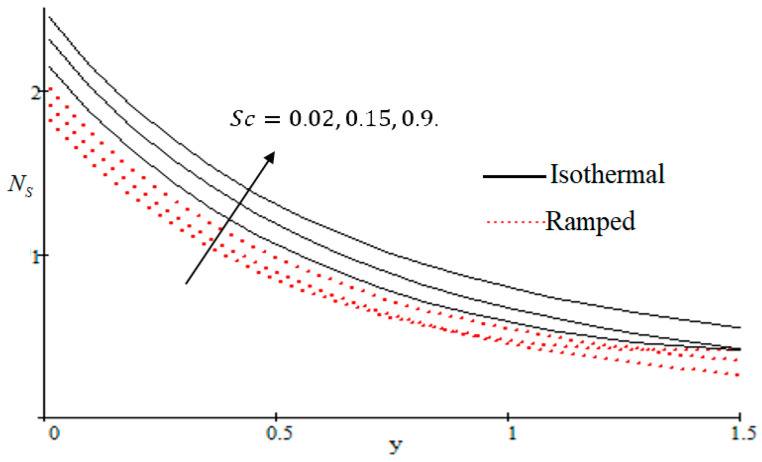
Influence of Sc on Ns.

**Figure 17:**
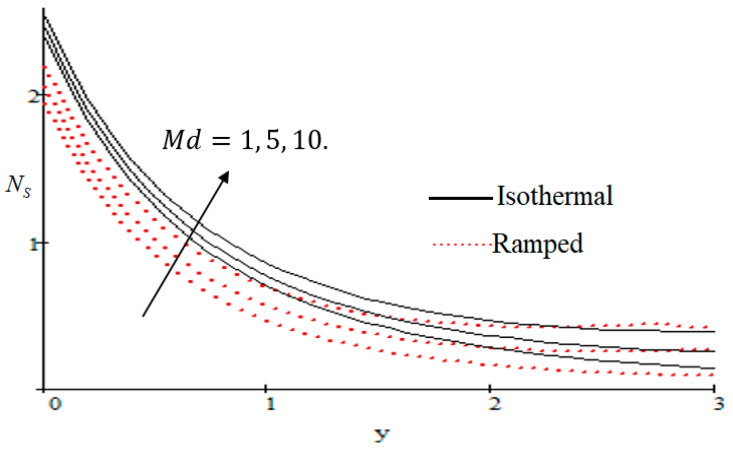
Influence of $M_{d}$ on Ns.

**Figure 18:**
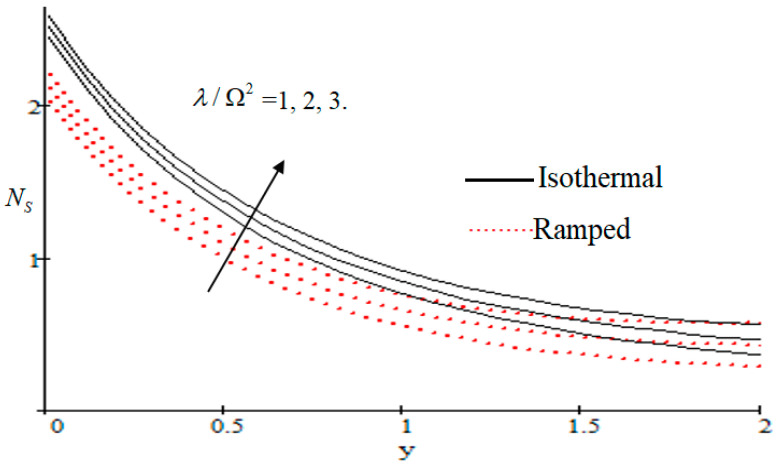
Influence of $\lambda /\Omega^{2}$ on Ns.

Properties of embedded parameters on $N_{S}$ are highlighted in Figure 9, Figure 10, Figure 11, Figure 12, Figure 13, Figure 14, Figure 15, Figure 16, Figure 17 and Figure 18. The effect of Grashof number Gr is shown in Figure 9. It is seen that $N_{S}$ increases with the increasing value of Gr. The influence of wall shear stress f is presented in Figure 10, where it is noticed that $N_{S}$ decreases with increasing f, therefore the rate of entropy generation can be reduced by increasing the value of f. In Figure 11, it is noticed that $N_{S}$ decreases after increasing the value of ${\mathrm{Pr}}_{eff}$. The effect of group parameter $B_{r}/\Omega$ over $N_{S}$ is highlighted in Figure 12, where it is observed that $N_{S}$ is the increasing function of group parameter $B_{r}/\Omega$. The graphical result shows that group parameter has an important ability to control $N_{S}$. The Brinkman group parameter $B_{r}/\Omega$ regulates significance of viscous effects and it is also noticed that this parameter is associated with the fluid viscosity term. The brinkman group parameter $B_{r}/\Omega$ appears directly proportional to the square of the velocity and an increase in it evidently accelerates flow and as a result entropy will increase. The effect of inverse permeability parameter $K_{p}$ is shown in Figure 13. It is seen that a decrease in $N_{S}$ occurs with an increase in $K_{p}$ for both ramped and isothermal walls temperature. The reason behind this fact is that the entropy generation is an increasing function of dissipative forces. Further, the impact of $K_{p}$ is more prominent at the stretching boundary whereas the effects die out in the region away from the boundary. In Figure 14, the value of magnetic parameter M is increased with the increasing $N_{S}$. The increasing graph of Gm is shown in Figure 15. A rapid increase in $N_{S}$ occurs with the increasing value of Gm. The effect of the Schmit number Sc over the local entropy generation rate $N_{S}$ is highlighted in Figure 16. It is observed that $N_{S}$ increases with increasing value of Sc. The effect of mass diffusion parameter $M_{d}$ is shown in Figure 17. It is detected that when $M_{d}$ increased $N_{S}$ is also improved. In Figure 18, the influence of group parameter $\lambda /\Omega^{2}$ over $N_{S}$ is shown, which an increasing function of the group parameter.

### 6.3. Influences of Embedded Parameters on Bejan Number

**Figure 19:**
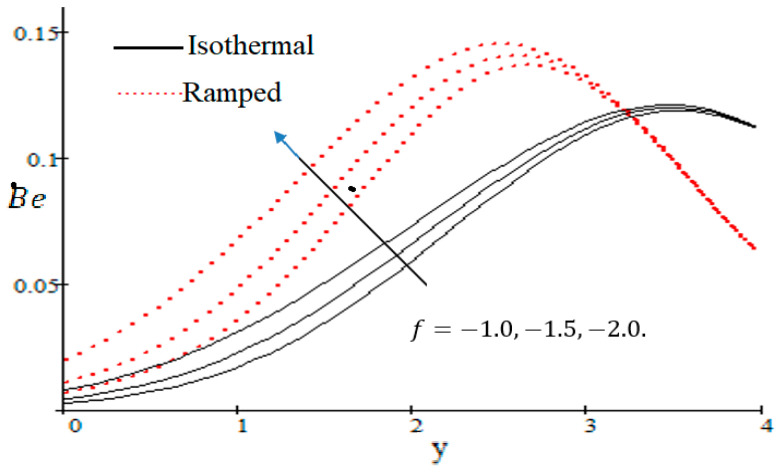
Influence of f on Be.

**Figure 20:**
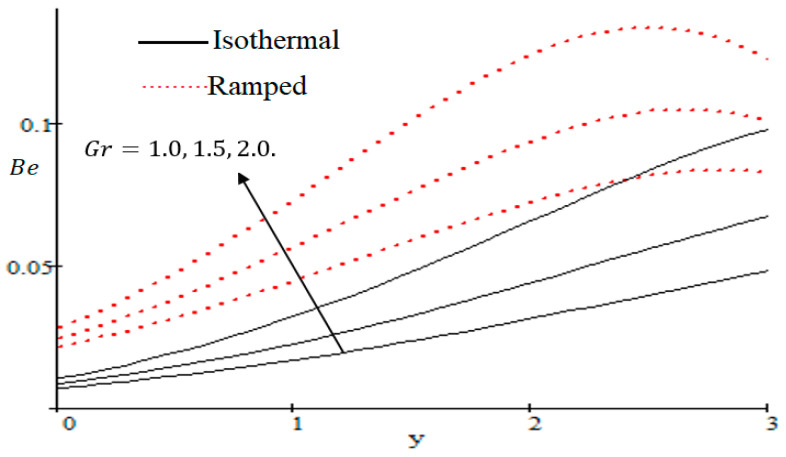
Influence of Gr on Be.

**Figure 21:**
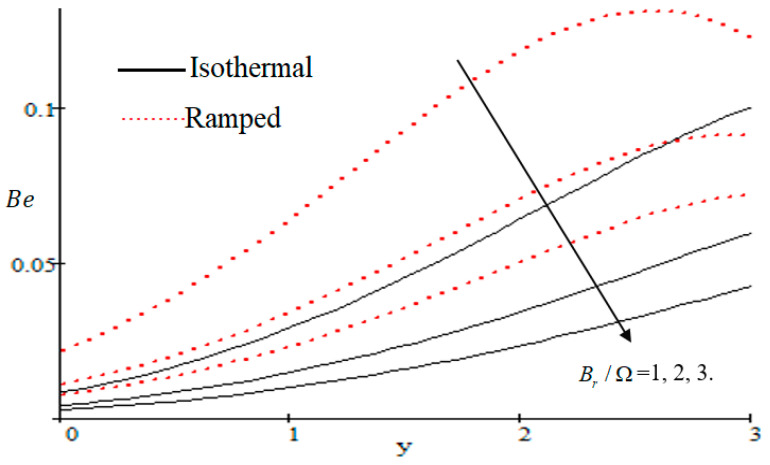
Influence of $B_{r}/\Omega$ on Be.

**Figure 22:**
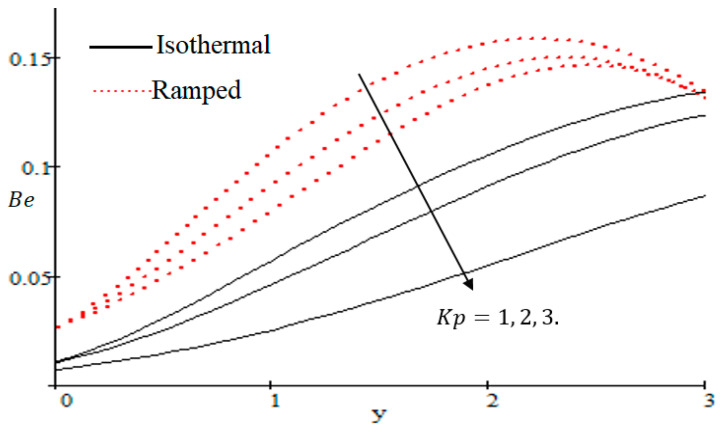
Influence of $K_{p}$ on Be.

**Figure 23:**
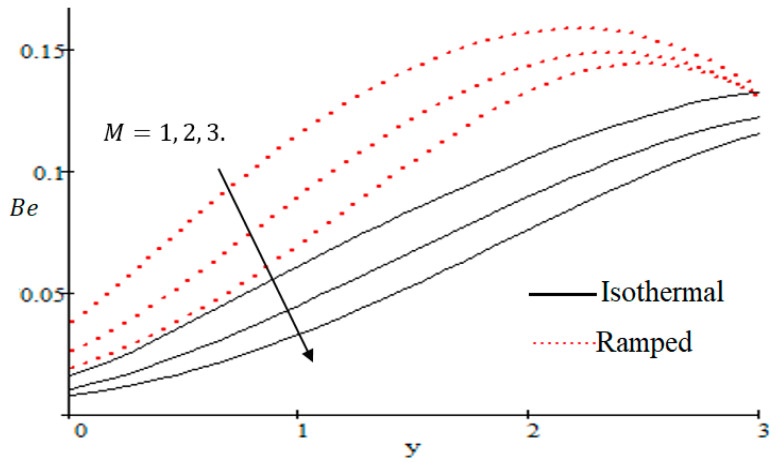
Influence of M on Be.

**Figure 24:**
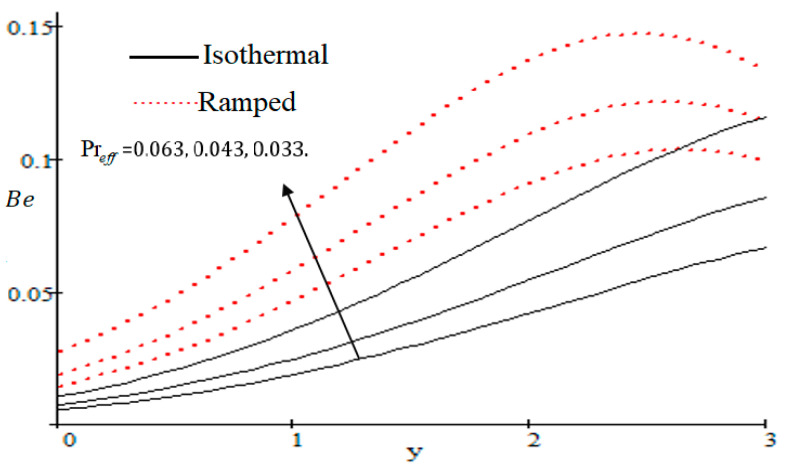
Influence of ${\mathrm{Pr}}_{eff}$ on Be.

**Figure 25:**
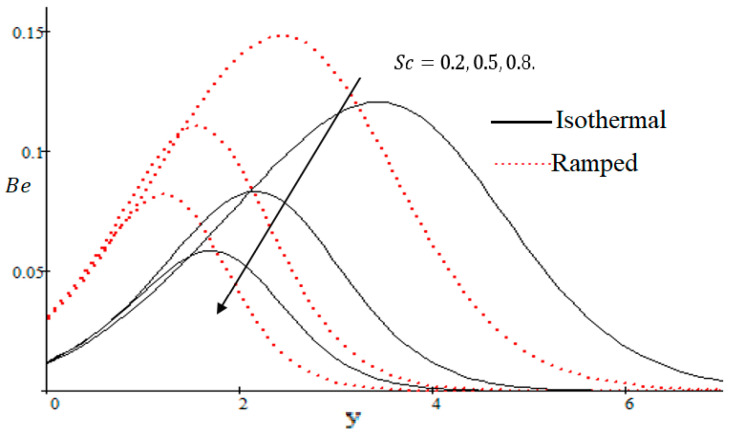
Influence of Sc on Be.

**Figure 26:**
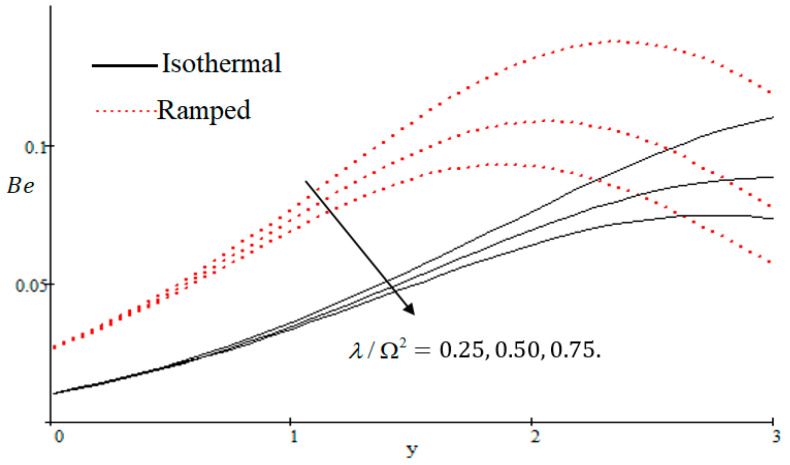
Influence of $\lambda /\Omega^{2}$ on Be.

**Figure 27:**
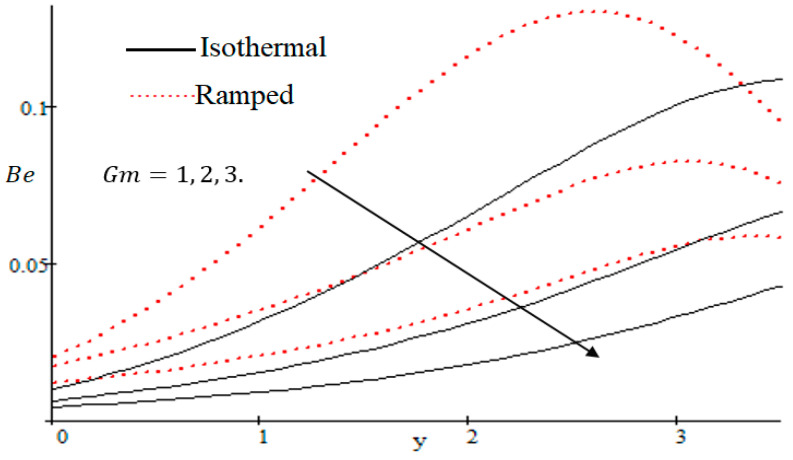
Influence of Gm on Bejan number.

**Figure 28:**
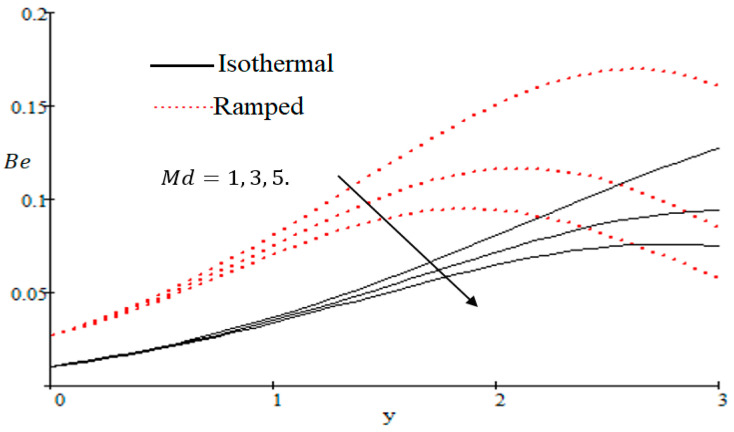
Impact of $M_{d}$ on Bejan number.

The Bejan number is quite useful, as one can get evidence about dominancy of magnetic field and fluid friction through heat transfer entropy or vice versa. The influence of f on the Bejan number is shown in Figure 19. The magnetic field leads to increasing the fluid friction and entropy with a decreasing value of f. The heat transfer reunification comes to be dominant in the region near to the plate with an increasing value of Gr, shown in Figure 20, while far away from the plate the friction of the fluid’s irreversibility become powerful and hence the Bejan number is strengthened. Figure 21 showed that the fluid friction entropy and the magnetic field are improved with an increase in the group consideration $B_{r}/\Omega$. Figure 22 explains that the Bejan number because of the magnetic field and the fluid friction becomes minor with an increase in the $K_{p}$ nearby plate. In Figure 23 it is showed that the fluid friction entropy and magnetic field increased with a rise in the value of M, for individually ramped and isothermal plates. Figure 24 showed an increase in the Bejan number with a decrease in ${\mathrm{Pr}}_{eff}$. The effect of the Schmit number Sc is showed in Figure 25, where it is observed that the Bejan number is decreasing with an increasing value of Sc. The effect of group parameter $\lambda /\Omega^{2}$ over the Bejan number is showed in Figure 26. It is noted that Be decreases with an increase in $\lambda /\Omega^{2}$. In Figure 27 the influence of Gm is shown and it is observed that Be decreases with an increase in Gm. The effect of $M_{d}$ is exposed in Figure 28, where it is seen that the Bejan number decreases with an increasing value of $M_{d}$.

## 7. Assumptions and Deductions

The effect of entropy generation for conjugate MHD unsteady flow through a porous medium near a vertical plate is deliberated. The exact solution for velocity profile is found by using the Laplace transform method. The Bejan number Be and number of local entropy generation Ns are discussed for various parameters. The effects are displayed for different embedded parameters. The main conclusions are:

- Rises in, $B_{r}/\Omega$, Sc, Gm, $\lambda /\Omega^{2}$ Md and M leads to decreases of the Bejan number for isothermal and ramped wall temperature individually.
- The Entropy generation in the fluid can be controlled and reduced by the f constant wall shear stress.
- Rises in $B_{r}/\Omega$, Sc, $M_{d}$, Pr, Gr, M, $K_{p}$ and Gm increase *Ns*.
